# Inflammatory conditions play a role in recurrence of PSC after liver transplantation: An international multicentre study

**DOI:** 10.1016/j.jhepr.2022.100599

**Published:** 2022-10-01

**Authors:** Thijmen Visseren, Nicole S. Erler, Julie K. Heimbach, John E. Eaton, Nazia Selzner, Aliya Gulamhusein, Frans van der Heide, Robert J. Porte, Bart van Hoek, Ian P.J. Alwayn, Herold J. Metselaar, Jan N.M. IJzermans, Sarwa Darwish Murad

**Affiliations:** 1 Erasmus MC Transplant Institute, Department of Gastroenterology and Hepatology, Erasmus MC University Medical Center Rotterdam, Rotterdam, The Netherlands; 2 Erasmus MC Transplant Institute, Department of Surgery, Division of Hepatopancreaticobiliary and Transplant Surgery, Erasmus MC University Medical Center Rotterdam, Rotterdam, The Netherlands; 3 Department of Biostatistics, Erasmus MC University Medical Center Rotterdam, Rotterdam, The Netherlands; 4 Department of Epidemiology, Erasmus MC University Medical Center Rotterdam, Rotterdam, The Netherlands; 5 Department of Surgery, Mayo Clinic College of Medicine and Science, Rochester, MN, USA; 6 Division of Gastroenterology and Hepatology, Mayo Clinic, Rochester, MN, USA; 7 Multiorgan Transplant Program, University Health Network, University of Toronto, Toronto, ON, Canada; 8 Department of Gastroenterology and Hepatology, University of Groningen and University Medical Centre Groningen, Groningen, The Netherlands; 9 HPB and Liver Transplant Surgery, University of Groningen and University Medical Centre Groningen, Groningen, The Netherlands; 10 LUMC Transplant Center, Department of Gastroenterology and Hepatology, Leiden University Medical Center, Leiden, The Netherlands; 11 Department of General Surgery, Nova Scotia Health Authority, Dalhousie University, Halifax, NS, Canada; 12 LUMC Transplant Center, Department of Surgery, Leiden University Medical Center, Leiden, The Netherlands

**Keywords:** Liver transplantation, Primary sclerosing cholangitis, Cholestatic liver disease, Recurrence of disease, Risk factors, Colectomy, Inflammatory bowel disease, ACR, acute cellular rejection, CCA, cholangiocarcinoma, CD, Crohn's disease, DBD, donation after brain death, DCD, donation after cardiac death, ELTR, European Liver Transplant Registry, HR, hazard ratio, IBD, inflammatory bowel disease, IBD-U, inflammatory bowel disease–unclassified, LD, living donor, LT, liver transplantation, MELD, model for end-stage liver disease, MRI, magnetic resonance imaging, PSC, primary sclerosing cholangitis, rPSC, recurrence of primary sclerosing cholangitis, SES-CD, Simple Endoscopic Score for Crohn's Disease, UC, ulcerative colitis

## Abstract

**Background & Aims:**

Liver transplantation (LT) for primary sclerosing cholangitis (PSC) is complicated by recurrence of PSC (rPSC) in up to 25% of recipients. Recurrence has been shown to be detrimental for both graft and patient survival. For both PSC and rPSC, a medical cure is not available. To predict and ideally to prevent rPSC, it is imperative to find risk factors for rPSC that can be potentially modified. Therefore, we aimed to identify such factors for rPSC in a large international multicentre study including 6 centres in PSC-prevalent countries.

**Methods:**

In this international multicentre, retrospective cohort study, 531 patients who underwent transplantation for PSC were included. In 25% of cases (n = 131), rPSC was diagnosed after a median follow-up of 6.72 (3.29–10.11) years post-LT.

**Results:**

In the multivariable competing risk model with time-dependent covariates, we found that factors representing an increased inflammatory state increase the risk for rPSC. Recurrent cholangitis before LT as indication for LT (hazard ratio [HR] 3.6, 95% CI 2.5–5.2), increased activity of inflammatory bowel disease after LT (HR 1.7, 95% CI 1.08–2.75), and multiple acute cellular rejections (HR: non-linear) were significantly and independently associated with an increased risk of rPSC. In contrast to the findings of previous studies, pretransplant colectomy was not found to be independently protective against the development of rPSC.

**Conclusions:**

An increased inflammatory state before and after LT may play a causal and modifiable role in the development of rPSC. Pretransplant colectomy did not reduce the risk of rPSC *per se*. Recurrent cholangitis as indication for LT was associated with an increased risk of rPSC.

**Impact and implications:**

Recurrence of PSC (rPSC) negatively affects survival after liver transplant (LT). Modifiable risk factors could guide clinical management and prevention of rPSC. We demonstrate that an increased inflammatory state both before and after LT increases the incidence of rPSC. As these are modifiable factors, they could serve as targets for future studies and therapies. We also added further evidence to the ongoing debate regarding preventive colectomy for rPSC by reporting that in our multicenter study, we could not find an independent association between colectomy and risk of rPSC.

## Highlights

• rPSC occurred after LT in 25% of recipients in this multicentre study from 6 transplant centres (Europe and North America).
• An increased systemically inflammatory state, both before and after LT, increases the risk of rPSC development.
• Increased IBD activity post-LT was associated with a higher risk of rPSC development.
• Performing a colectomy before LT was not associated with a reduction of rPSC.

## Graphical abstract

**Figure:**
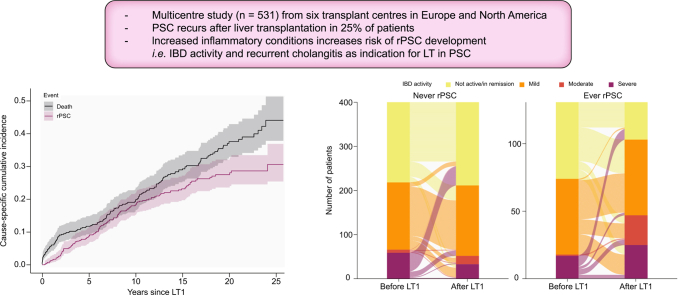

## Introduction

Primary sclerosing cholangitis (PSC) is a chronic and progressive biliary disease that results in destructed intrahepatic and extrahepatic bile ducts.^1^ Globally, the prevalence of PSC is found to be the highest in northern parts of both the European and American continents with 6–16 cases per 100,000 inhabitants[2], [3], [4] and therefore is an important indication for LT in these regions.^5^

PSC is often accompanied by 1 or more episodes of cholangitis, inducing fibrosis and cirrhosis, and an enhanced risk of both cholangiocarcinoma (CCA) and colorectal carcinoma.^2^ The co-existence of inflammatory bowel disease (IBD) is striking with percentages of up to 85%, most often characterised as PSC-IBD of the type resembling ulcerative colitis (UC).^6^ There is no medical cure available, and liver transplantation (LT) is indicated in individuals with end-stage liver disease or with multiple and life-threatening episodes of cholangitis.^7^ A third category of potential transplant candidates are individuals with PSC and perihilar CCA.^8^

Partly owing to their relatively young age at transplant, individuals with PSC have, in general, a favourable long-term outcome after LT.^9^ A major drawback is recurrence of PSC (rPSC), observed in up to 25% of patients.^10^ Recurrence is diagnosed in most individuals within 5 years after transplantation with the identical symptoms of the primary disease. The effects of rPSC on long-term survival have been studied extensively, and both graft and patient survival have been shown to be impacted negatively. Moreover, rPSC leads more frequently to retransplantation and hence adds an additional burden on the existing scarcity of donor livers.^11^ The aetiopathogenesis of rPSC has been studied in multiple cohorts but without consistent results, besides that several aspects of IBD are involved in risk for rPSC.^12^

In 2018, a meta-analysis involving 14 studies on risk factors for rPSC was reported.^13^ In this study, several risk factors were found to be associated with an increased risk of rPSC, such as the presence of IBD, cholangiocarcinoma before LT, donor age (per 10 years), any episode of acute cellular rejection (ACR), multiple episodes of ACR, and laboratory model for end-stage liver disease (MELD) score. The only factor associated with a decreased risk of rPSC was colectomy before LT. None of these factors were examined in all included studies, a limitation addressed by the authors; hence, no interdependence between factors or independency of the observed associations could be examined further. Moreover, none of the published studies so far have shown compelling evidence strong enough to recommend a colectomy as a preventive strategy.

We performed an international study in patients who underwent transplantation for PSC in 6 transplant centres from PSC-prevalent countries to analyse risk factors for rPSC with the aim of identifying potentially modifiable factors to alter or avoid the development of rPSC.

## Patients and methods

### Patient data collection

Six liver transplant centres participated in this retrospective study. To ensure a sufficient follow-up period, we included all patients who underwent transplantation for PSC between 1990 and 2005. To ensure uniform data collection, a single member of the research group from the initiating centre visited all the participating transplant centres, based in the United States of America (n = 1), Canada (n = 2), and The Netherlands (n = 3). Data were captured anonymously using a predefined case report form. Data were subsequently stored in an online database with an audit trail and server protection. Data were collected on pre-, peri-, and post-transplant parameters. We focused on donor and recipient characteristics, transplant procedure characteristics, and IBD characteristics.

The exclusion criteria were formulated as follows: (1) paediatric liver transplant recipients (*i.e.* age <18 years); (2) ABO-incompatible transplants; and (3) if pre- or post-transplant data were completely lacking; in all other cases, we used the information available.

### Ethical considerations

The retrospective study design was approved by the medical ethical board of the initiating centre (MEC-2014-060), as well as the ethical boards of the participating centres. Data transfer agreements were signed to allow data transfer to the initiating centre for analysis purposes.

### Diagnosis of rPSC

The established Mayo criteria were strictly applied to identify the patients affected by rPSC (Box 1). These criteria are based on radiological diagnosis of a stricturing cholangiopathy alike PSC appearing for the first time at least 90 days after LT, in the absence of other known causes (*i.e.* ischaemic cholangiopathy or portal biliopathy) of secondary sclerosing cholangiopathy.^14^ We evaluated all MRI and biopsy reports, including the histology of the explant liver to confirm the PSC diagnosis before transplant. In case of doubt, we consulted an expert radiologist (RSD) or pathologist (MD) to confirm the most likely diagnosis (see Acknowledgements). Our interpretation of the available evidence was always considered conclusive, in case of a conflict with the available diagnosis present in the medical charts.

Box 1 **The Mayo criteria for rPSC after LT as postulated by Graziadei et al.14 in 1999.**

**Figure:**
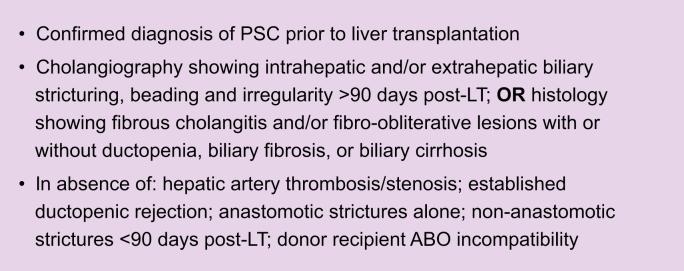

LT, liver transplantation; PSC, primary sclerosing cholangitis; rPSC, recurrence of primary sclerosing cholangitis.

### Risk factors for rPSC – definitions

The main purpose of this study was to evaluate potential modifiable risk factors. We selected the covariate panel based on previous studies and added the indication for transplantation as an additional risk factor as suggested in an earlier single-centre study.^15^

The stages of IBD activity were indexed, both before and after LT, according to endoscopic and microscopic inflammatory activity, clinical symptoms described, and the (medical) treatment patients received. We used a combination of these indicators to index individuals with UC and Crohn’s disease (CD) into 4 categories: (1) no (active) IBD or in remission, (2) mild, (3) moderate, and (4) severe. For individuals with UC, the endoscopic Mayo score was leading for the classification.^16^ For individuals with CD, we used the Simple Endoscopic Score for Crohn’s Disease (SES-CD) score,^17^ with the following cut-offs: inactive (0–2), mild (3–6), moderate (7–15), and severe (>15). When these scores were not present or extractable from endoscopy reports, other indicators were used to classify the patients as follows: those requiring a steady dose of maintenance medication without any flares were classified as mild; those with up to 2 flares requiring an induction dose were classified as moderate; and those with a flare requiring hospitalisation or an untreatable IBD requiring colectomy were classified as severe. These criteria are in line with a recent report regarding classification of IBD severity.^18^

The indexed stages of IBD activity were analysed using a variable indicating whether the activity was decreased, stable, or increased after LT, compared with before LT. Moreover, we included whether a patient underwent a total proctocolectomy before LT.

The indication for the initial LT was also considered to be a potential risk factor, and 3 patient categories were defined: (1) recurrent cholangitis, (2) end-stage liver disease, and (3) perihilar CCA. The first category includes individuals with recurrent cholangitis (at least twice per half year) unresponsive to endoscopic treatment, and the second category includes individuals with decompensated cirrhosis. The third category includes individuals with a perihilar CCA before LT, including patients diagnosed during the transplant procedure.

ACR was collected with the corresponding date of diagnosis (liver biopsy), and chronic cellular rejection was collected separately in a binary (yes/no) variable.

### Statistics

Patient and donor characteristics were summarised for all patients (after exclusions) as well as divided into groups of patients that did or did not experience rPSC using medians and 2.5% and 97.5% quantiles for continuous variables and frequency and proportions for categorical variables.

The cause-specific (rPSC or death) cumulative incidences were determined using the Aalen–Johansen estimator.

To investigate the associations of several potential risk factors with the hazard of rPSC, considering the competing risk of death, we fitted a Cox proportional hazards model. The model included the recipient’s age at the first LT, type of donor (donation after brain death [DBD], donation after cardiac death [DCD], or living donor [LD]), change in IBD activity (decreased, stable, or increased), IBD activity after the first LT (not active/in remission, mild, moderate, or severe), whether a total proctocolectomy had been performed, the indication for the first LT (cirrhosis, recurrent cholangitis, or perihilar CCA), the cumulative number of ACRs, and whether chronic ACR was present. Patients were censored at the date of last clinical follow-up.

The type of donor and cumulative number of ACRs were included as time-varying covariates, where the donor type changed at each transplant and the number of ACRs increased at each ACR. Moreover, to allow for a non-linear shape of the association between the number of ACRs and risk for rPSC or death, we modelled this variable using a natural cubic spline with 2 degrees of freedom. Results from the multivariable Cox model are presented as the hazard ratio (HR) with corresponding 95% CIs and *p* values. Analyses were performed in R version 4.0.5^19^ using the survival package (version 3.2-13; R Foundation for Statistical Computing, Vienna, Austria).^20^

## Results

In total, 546 patients who received a first LT for PSC were included. Fifteen (2.7%) patients were excluded because the medical records before or after LT were missing (*e.g.* when a patient moved to another region before or after transplant and no information was available). The characteristics of the remaining 531 patients are shown in Table 1. Most of the patients are male (n = 359; 68%), the median age at the first LT was 45.7 (22.9–64.4) years, and 419 (79%) patients were diagnosed with IBD at any point in time. Of them, 355 (85%) had UC, 51 (12%) CD, and 13 (3%) IBD–unclassified (IBD-U). The indication for the first LT was recurrent cholangitis in 110 (21%) patients, end-stage liver disease in 386 (73%), and CCA in 35 (6%) patients. Of all patients who underwent transplantation, 105 (20%) received 1 retransplant and 9 (2%) received 2 retransplants. Three patients received 3 retransplants, and 1 patient received 4 retransplants. The main causes of retransplantation were rPSC (n = 37; 31%), vascular complications (n = 22; 19%), and (not rPSC-related) biliary complications (n = 19; 16%).

**Table 1:** **Baseline characteristics of 531 individuals with PSC receiving a liver transplant between 1990 and 2005 in the 6 contributing transplant centres**.

	Total	rPSC at any time	Never rPSC
	N = 531	n = 131	n = 400
**Recipient characteristics (first LT)**			
Recipient sex			
Male	359 (67.6%)	92 (70.2%)	267 (66.8%)
Female	172 (32.4%)	39 (29.8%)	133 (33.2%)
Recipient age at LT	45.7 [22.9, 64.4]	42.1 [20.8, 61.6]	47.1 [25.2, 64.6]
IBD			
No IBD	112 (21.1%)	16 (12.2%)	96 (24.0%)
UC	355 (66.9%)	100 (76.3%)	255 (63.7%)
CD	51 (9.6%)	12 (9.2%)	39 (9.8%)
IBD–unclassified	13 (2.4%)	3 (2.3%)	10 (2.5%)
Total colectomy			
Before LT	55 (10.4%)	10 (7.6%)	45 (11.2%)
After LT	55 (10.4%)	20 (15.3%)	35 (8.8%)
Indication			
End-stage liver disease	386 (72.7%)	62 (47.3%)	324 (81.0%)
Recurrent cholangitis	110 (20.7%)	63 (48.1%)	47 (11.8%)
Perihilar CCA	35 (6.6%)	6 (4.6%)	29 (7.2%)
MELD at LT	16.0 [8.0, 28.0]	15.0 [8.0, 25.5]	16.0 [8.0, 29.7]
Missing	154 (29.0%)	41 (31.3%)	113 (28.2%)
Donor type			
DBD	454 (85.5%)	120 (91.6%)	334 (83.5%)
DCD	34 (6.4%)	7 (5.3%)	27 (6.8%)
LD	43 (8.1%)	4 (3.1%)	39 (9.8%)
Warm ischaemic time	56.0 [27.0, 96.9]	58.0 [32.6, 98.4]	56.0 [26.4, 94.4]
Missing	108 (20.3%)	17 (13.0%)	91 (22.8%)
Cold ischaemic time	478.0 [131.2, 819.0]	485.5 [249.6, 747.0]	475.0 [128.0, 838.6]
Missing	115 (21.7%)	19 (14.5%)	96 (24.0%)
Biliary anastomosis			
Duct-to-duct	58 (11.6%)	14 (11.4%)	44 (11.6%)
Roux-en-Y	443 (88.4%)	109 (88.6%)	334 (88.4%)
Missing	30 (5.6%)	8 (6.1%)	22 (5.5%)

CCA, cholangiocarcinoma; CD, Crohn’s disease; DBD, donation after brain death; DCD, donation after cardiac death; IBD, inflammatory bowel disease; LD, living donor; LT, liver transplantation; MELD, model for end-stage liver disease; PSC, primary sclerosing cholangitis; rPSC, recurrence of primary sclerosing cholangitis; UC, ulcerative colitis. Values are represented in N (%) for categorical and median [2.5% and 97.5% quantiles] for continuous variables.

**Fig. 1:**
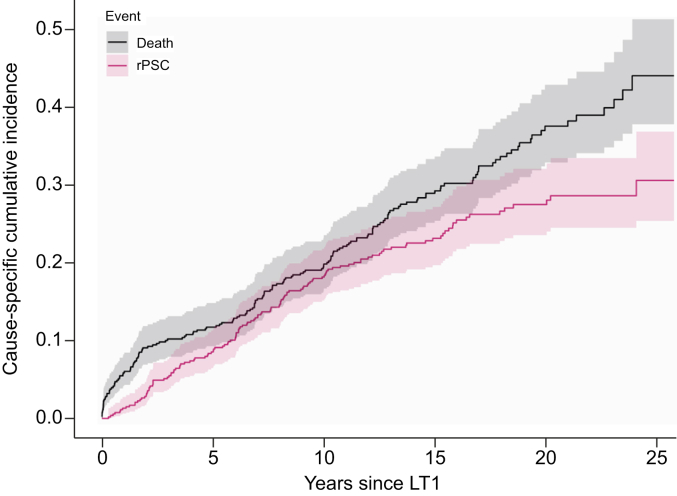
Cumulative incidence (with 95% CI) for rPSC and recipient death. Naturally, a gradual increase of recipient death is observed over the years, whereas the cumulative incidence curve of rPSC is flattening after 15 years of follow-up. LT, liver transplantation; rPSC, recurrence of primary sclerosing cholangitis.

At the end of follow-up, considering all (re)transplants, rPSC was diagnosed in 131 (25%) patients after a median time of 6.72 (3.29–10.11) years after the first LT. In total, 318 (60%) patients were alive with a median follow-up of 15.24 (12.00–19.86) years, and 213 (40%) patients died after a median of 8.81 (2.87–13.99) years after the first LT (Fig. 1). The main causes of death were malignancies (n = 45; 21%), infections (n = 29; 14%) and graft failure related (n = 9; 4%).

### Risk factors for rPSC and recipient death

To analyse the risk factors for rPSC, we constructed a multivariable Cox model, with consideration of the competing risk of death. The results from this model are shown in Table 2, Table 3 and show the HR for both rPSC (Table 2) and recipient death (Table 3). Of note, we did not find an association between immunosuppressive regimen (cyclosporine based *vs*. tacrolimus based *vs*. other), early graft dysfunction or ischaemia times on risk of rPSC, or death (data not shown).

**Table 2:** **Multivariable competing risk Cox proportional hazards model for rPSC**.

Variable	rPSC			
	HR	2.5%	97.5%	*p* value
Age at first LT (per 1-year increase)	0.995	0.979	1.011	0.540
Donor type				
DBD donor	Ref.			
DCD donor	0.502	0.206	1.225	0.130
LD	0.388	0.149	1.011	0.053
IBD activity post-LT as compared with pre-LT				
Stable	Ref.			
Decreased	0.886	0.389	2.017	0.772
Increased	1.730	1.087	2.755	0.021
IBD activity post-LT				
Not active	Ref.			
Mild	1.696	0.960	2.997	0.069
Moderate	2.335	1.126	4.839	0.023
Severe	1.270	0.573	2.817	0.556
Proctocolectomy before LT	1.568	0.535	4.593	0.412
Indication first LT				
End-stage liver disease	Ref.			
Recurrent cholangitis	3.584	2.451	5.240	0.000
Perihilar CCA	2.497	0.992	6.285	0.052
ACR	∗			
Chronic cellular rejection	0.417	0.052	3.370	0.412

Results are expressed in HR with respective 95% confidence intervals. ACR, acute cellular rejection; CCA, cholangiocarcinoma; DBD, donation after brain death; DCD, donation after cardiac death; HR, hazard ratio; IBD, inflammatory bowel disease; LD, living donor; LT, liver transplantation; rPSC, recurrence of primary sclerosing cholangitis.

**Fig. 2:**
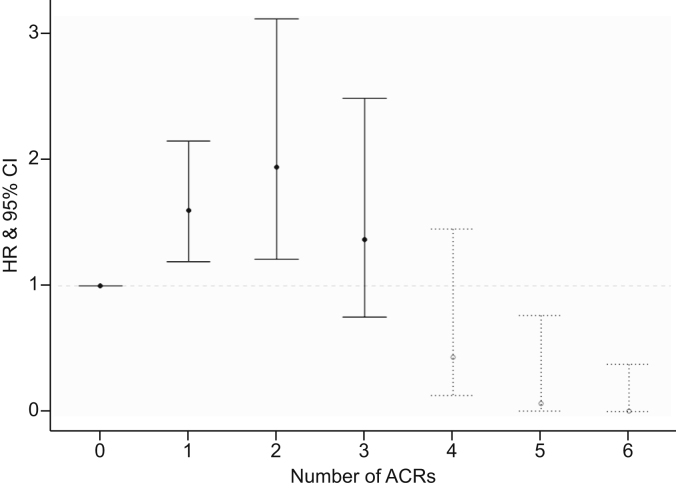
ACR and risk of rPSC. Results are expressed in HR with respective 95% confidence intervals. The association between multiple ACRs and rPSC is non-linear and is therefore not represented by a single *p* value. Individuals with 1 or 2 ACRs are incremental at higher risk for rPSC, whereas 3 ACRs are not associated statistically significant with an increased risk. As 4 or more ACRs in 1 patient is scarce, this is less informative and therefore displayed in grey. This graph is derived from the multivariable competing risk Cox proportional hazards model for rPSC. ACR, acute cellular rejection; HR, hazard ratio; rPSC, recurrence of primary sclerosing cholangitis.

∗ Non-linear HR; please see corresponding Fig. 2 for HR estimation for ACR.

**Table 3:** **Multivariable competing risk Cox proportional hazards model for recipient death**.

Variable	Death			
	HR	2.5%	97.5%	*p* value
Age at first LT (per 1-year increase)	1.039	1.023	1.056	0.000
Donor type				
DBD donor	Ref.			
DCD donor	2.846	1.699	4.768	0.000
LD	0.630	0.333	1.193	0.156
IBD activity post-LT as compared with pre-LT				
Stable	Ref.			
Decreased	0.700	0.306	1.603	0.399
Increased	0.746	0.426	1.307	0.305
IBD activity post-LT				
Not active	Ref.			
Mild	0.663	0.458	0.960	0.030
Moderate	0.616	0.250	1.520	0.293
Severe	0.536	0.235	1.224	0.139
Proctocolectomy before LT	1.123	0.437	2.883	0.810
Indication first LT				
End-stage liver disease	Ref.			
Recurrent cholangitis	0.603	0.364	0.998	0.049
CCA	1.748	1.003	3.047	0.049
Acute cellular rejection	∗			
Chronic cellular rejection	2.454	1.198	5.027	0.014

Results are expressed in HR with respective 95% confidence intervals. CCA, cholangiocarcinoma; DBD, donation after brain death; DCD, donation after cardiac death; HR, hazard ratio; IBD, inflammatory bowel disease; LD, living donor; LT, liver transplantation.

∗ Non-linear HR.

### Indication for LT

Patients who were listed for recurrent cholangitis had a 3.6-fold increased risk of rPSC (95% CI 2.5–5.2; *p* = 0.000) compared with patients who underwent transplantation for decompensated cirrhosis (Table 2). Of the 110 (21%) patients who underwent transplantation for recurrent cholangitis, 63 (57%) were diagnosed with rPSC after a median of 7.21 (3.94–10.11) years as compared with 62 (16%) cases in patients who underwent transplantation for cirrhosis (n = 386; 73%) after a median of 6.40 (2.44–10.57) years. Patients who underwent transplantation for perihilar CCA (n = 35; 6%), were diagnosed with rPSC in 6 (17%) cases after a median of 3.94 (2.29–7.25) years. Of the 110 patients who underwent transplantation for recurrent cholangitis, 20 (18%) showed signs of cirrhosis in their explant liver. Of these 20 patients, 3 (15%) were diagnosed with rPSC.

Survival after LT is also influenced by the indication for the first LT (Table 3). Patients who underwent transplantation for recurrent cholangitis had the best survival of the 3 different patient categories with an HR of 0.6 (95% CI 0.36–0.99; *p* = 0.049) for recipient death. Patients who underwent transplantation for CCA had the worst outcome with an increased risk of death of 1.7 (95% CI 1.–3.05; *p* = 0.049). The median time to death for the indications recurrent cholangitis (n = 40), cirrhosis (n = 159), and CCA (n = 14) was 10.05 (6.42–18.26), 8.61 (2.53–13.59), and 2.73 (1.41–10.07) years, respectively.

### Acute and chronic cellular rejection

The effect of ACRs on the risk of rPSC appeared to be non-linear and is therefore not represented by 1 HR with a corresponding *p* value. Instead, the results are displayed in Fig. 2, which shows that 1 (n = 178; 34%) or 2 (n = 70; 13%) ACR episodes increases the risk of rPSC compared with no ACR. Because they are rare, we could not find evidence that 3 (n = 25; 5%) or more (n = 16; 3%) ACRs are associated with an even higher risk of rPSC.

Fourteen (3%) patients were diagnosed with chronic ACR. Only 1 of them was also diagnosed with rPSC. These 14 patients did show a 2.5-fold risk of death (95% CI 1.19–5.03; *p* = 0.014), in comparison with individuals without chronic ACR (Table 3).

### IBD and colectomy

Active IBD after LT was associated with rPSC (Table 2). Compared with those with inactive IBD, individuals with mild (n = 215), moderate (n = 41), and severe IBD (n = 58) had an increased risk of rPSC with HRs of 1.7 (95% CI 0.96–2.99; *p* = 0.069), 2.3 (95% CI 1.12–4.83; *p* = 0.023), and 1.3 (95% CI 0.57–2.81; *p* = 0.556), respectively. There was no evidence for an association between active IBD and recipient death.

**Fig. 3:**
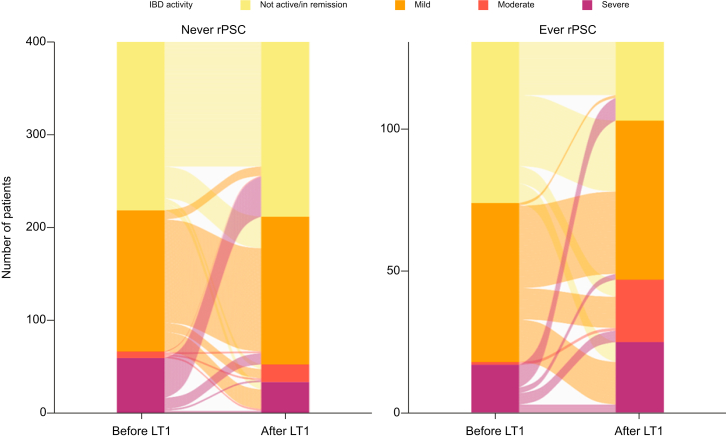
IBD activity before and after LT in individuals with and those without rPSC. The cohort is split into patients who did and those who did not develop rPSC. Each patient’s IBD state is shown before and after LT, and a line connects these states to show pattern differences in IBD state before and after LT. In patients diagnosed with rPSC, an increase of IBD activity after LT is observed, with a noticeable decrease in individuals without an active IBD. IBD, inflammatory bowel disease; LT, liver transplantation; rPSC, recurrence of primary sclerosing cholangitis.

Also, an increase in IBD activity after LT was associated with an increased risk of rPSC (HR 1.7, 95% CI 1.08–2.75; *p* = 0.021), compared with a stable IBD activity before and after LT. By contrast, a decrease in IBD activity after LT was not associated with a statistically significant decreased risk of rPSC (HR 0.9, 95% CI 0.4–2.0), as shown in Table 2. Fig. 3 visualises how IBD activity changed from before to after the first LT for patients who did (or did not) experience rPSC at any given time. It demonstrates that, although the distribution of IBD activity categories remained relatively stable in individuals without rPSC, there was a significant increase in activity of IBD after LT in patients who did experience rPSC. Changes of IBD activity after LT did not significantly impact the risk of recipient death.

The colon was removed in 55 (10%) patients before the first LT. Of these patients, 10 (18%) were diagnosed with rPSC during the follow-up of this study. Table 2 shows that there was no evidence that a removed colon influences the risk of rPSC (*p* = 0.412). After the first LT, the colon was removed in (coincidentally also) 55 (10%) patients, for various reasons (*e.g.* IBD or [suspected] colorectal carcinoma). Of those 55 patients, 20 (36%) developed rPSC, and the remaining 35 (64%) did not.

### Age and donor type

Age at the first LT did not seem to impact the risk of rPSC (Table 2). Although no difference was found for DBD or DCD donors, results (albeit not statistically significant) suggested a lower risk of rPSC (HR 0.39, 95% CI 0.14–1.01; *p* = 0.053) for patients receiving a liver from an LD.

Age at the first LT was associated with an increased risk of recipient death, with a 1.04-fold increase for each incremental year after LT (95% CI 1.02–1.05; *p* = 0.000). Patients who had received a DCD had an increased risk of recipient death as well, with an HR of 2.8 (95% CI 1.69–4.76; *p* = 0.000), compared with patients receiving a DBD (Table 3). The median time to death after LT was 7.2, 8.9, and 10.2 years, for DCD, DBD, and LD, respectively. In case of a retransplant, the median time to re-LT after LT with a DCD, DBD, or LD was 0.53, 3.7, and 9.4 years, respectively.

## Discussion

In this international multicentre study in PSC-prevalent countries, we found that individuals with PSC who underwent transplantation for recurrent cholangitis are more at risk of developing rPSC than individuals with PSC who underwent transplantation for end-stage liver disease. We also showed that an increase in IBD activity after LT was associated with a higher risk of developing rPSC. The previously known increased risk by multiple ACRs is confirmed in our study. Performing a colectomy, however, was shown not to be protective, in contrast to findings of earlier studies.^21^

Since the original description of rPSC by Graziadei *et al.*^14^ in 1999, several studies have been performed to identify risk factors for rPSC and evaluate the outcome for graft and patient survival, often with conflicting results.[22], [23], [24], [25], [26], [27] Although recent studies have established a negative impact on graft and patient survival,^11^ the risk factors for rPSC remain not fully elucidated, with inconsistencies between studies, often depending on the available clinical parameters within the studied populations. These inconclusive results could furthermore be explained by the relatively low number of patients per study, short follow-up times (rPSC can develop many years post-LT), and most often a single-centre approach.[28], [29], [30], [31] Moreover, the statistical approaches of these studies were suboptimal as several authors did not include rPSC as a time-varying variable, used a combined endpoint (recipient death and graft loss), or did not consider the competing risk of recipient death.^26^^,^^32^ A meta-analysis including 14 studies with a total number of 2,159 patients identified 7 factors associated with rPSC^13^: cholangiocarcinoma, IBD, donor age, MELD score, ACR, and multiple ACRs were all associated with an increased risk for rPSC, and colectomy before transplantation was associated with a reduced risk for rPSC. However, in this meta-analysis using reported, not individual patient data, these factors were presented without their interdependent relationships, and hence, it is unclear to what extent these factors were indeed independently associated with rPSC. Also, none of the factors were present in all 14 reviewed studies, and at best 1 variable was studied in 10 out of 14 studies, further limiting the generalisability of the results and adding to the overall puzzlement. To further elucidate the risk factors for rPSC and aiming to include all potential confounders, we conducted this international multicentre study.

The rate of rPSC in our cohort of 531 patients was 25%, which is in line with earlier studies, including our recent report of the European Liver Transplant Registry (ELTR) database.^11^ In our study, the indication for LT was shown to be of major influence on the risk of rPSC. Patients who underwent transplantation for recurrent cholangitis were shown to have a 3.6-fold increased risk of developing rPSC, compared with patients who underwent transplantation with end-stage liver disease. Interestingly, the time to rPSC and the time to death were similar for the 2 patient groups. As far as we know, none of the other studies have investigated the role of LT indication in rPSC, and hence, this represents a novel finding. Although this knowledge may help in our understanding of the pathophysiologic mechanism of rPSC, it is important to note that this finding has limited clinical consequences as recurrent cholangitis is currently difficult to prevent.

This finding nevertheless made us speculate on the role of an active immune system (*i.e.* a high state of inflammation) in the development of rPSC. Indeed, we also found that an increased activity of IBD after transplantation was associated with a higher recurrence rate of PSC. Furthermore, even the rate of (at least 1) ACR in individuals with PSC who underwent transplantation of 34% was at the high end of ACR rates usually seen (10–25%) in other liver diseases in the tacrolimus era.^33^ For ACR, it should be noted that the effect seemed to dissipate after the second episode, possibly related to an assumed increased doses of immunosuppressive therapeutics given to treat the recurrent ACR. Chronic rejection, however, was rare and not predictive.

In broader terms, all these factors share the presence of an increased (auto)inflammatory state. Given the fact that we, and others, recently reported that rPSC after LT may be associated with specific changes in the gut microbiome pretransplant, which may trigger changes towards activation of the immune system,^15^ this speculation becomes more tempting. More importantly, these observations may support strategies to prevent or limit immune activations and the development of rPSC.

Along these lines, we found that colectomy before transplantation was not protective for rPSC development in the multivariable analysis, after correcting for IBD activity parameters. This finding seems in contrast to a Nordic study^23^ and the meta-analysis of Steenstraten *et al.*,^13^ who both concluded that colectomy before transplantation was associated with a reduced risk for rPSC. Noteworthy is that the meta-analysis data on colectomy were mainly driven by data from the same Nordic study and represented uncontrolled univariate analysis. However, our finding is in line with a large UK study^26^ and the work of Trivedi *et al.*,^34^ describing that individuals with and without a colectomy have a similar incidence of graft loss after LT for PSC. Also, we found that those with pretransplant severe activity and post-transplant mild activity (*i.e.* the decreased activity group) were not at increased risk of developing rPSC, whereas those with increasing severity after LT had a significantly higher risk. Although further data are needed to make more firm conclusions, 1 of the possible explanations for these findings could be that the IBD status post-transplant is more important for rPSC development than IBD activity before LT.

Our knowledge on the immune landscape of PSC is growing. New recent data have revealed the JAK-STAT pathway as a promising and targetable underlying mechanism that could be responsible for the (prolonged) activated inflammatory state in individuals with PSC.^35^ Multiple studies in IBD have demonstrated efficacy of several selective small-molecule JAK inhibitors, such as tofacitinib, which has been approved for the treatment of CU.^36^ These molecules may play a role in the prevention of rPSC by inhibiting the cascade of inflammatory response resulting in biliary inflammation.^37^ As we learn more about the pathogenesis of PSC and its connection with IBD, the roads are slowly being paved for future trials with small-molecule JAK inhibitors in the setting of (r)PSC, realising the increased risk of infections caused by immunosuppression being the leading cause of graft failure and patient death in individuals with PSC who underwent LT.^38^^,^^39^

In our study, recipient survival was found to be associated with recipient age, the use of DCD livers, pretransplant end-stage liver disease, pretransplant perihilar CCA, and chronic rejection, all of which were found to increase the risk of death significantly, whereas IBD activity did not seem to be strongly associated with recipient survival. The risk of recipient death was estimated to be 2.8-fold while using DCD livers, which is a surprising finding as a recent study showed the usage of DCD livers to be safe on the long term.^40^ Apparently, this is still a subject open for debate, and in our opinion, DCD livers should only be used if the liver is optimised using novel machine perfusion techniques.^41^ For perihilar CCA, it is known that survival may be poorer owing to cancer recurrence.^42^ Increased recipient age is a known risk factor of comorbidities and higher post-transplant mortality, and our result is in line with previous studies.^43^

A major strength of this study is the large number of patients included. With 531 patients who underwent transplantation for PSC, with a significant median follow-up of more than 15 years, this cohort is as large as the largest cohort (n = 565) studied so far regarding risk factors for rPSC by Ravikumar *et al.*^26^ Moreover, to ensure uniform data collection, all patient charts were reviewed on-site to collect data in a uniform manner. To confirm the diagnosis of PSC in the first explant, all cases were verified using the explant histological report. The international multicentre setting has reduced the risk of bias created by decisions made in a single centre. Our statistical approach is comprehensive and included multivariable analysis of several risk factors, in a time-varying setting if needed, including the competing risk of death. To safeguard scientific integrity, the results of the statistical analyses were blinded until the authors agreed on the model of choice, after which we did not allow any changes in variables included.

This study has limitations as well. First, it is known that the diagnosis of rPSC can be challenging, with secondary sclerosing cholangitis (*e.g.* ischaemic biliopathy) resulting in the same pathological changes on magnetic resonance cholangiopancreatography. By using the Mayo definition and hence excluding vascular or inflammatory causes of cholangiopathy, we tried to decide as uniform as possible, but without a gold standard, there will always be some room for error. Second, despite the significant size of our cohort, we were limited in terms of the total number of variables we could include in the statistical model to prevent overfitting. Analysis of the timing of rPSC diagnosis (*e.g.* early *vs*. late rPSC) regarding risk factors and the cause of death or re-LT seemed interesting but not feasible owing to small numbers. Third, the early transplants were not always fully documented, resulting in missing all pretransplant data in some cases, which left us no choice but to exclude these cases. Also, over the years, much has changed in terms of surgical techniques, immunosuppressive drugs, organ allocation, and imaging modalities. Nevertheless, we believe that the cohort is large enough to flatten out era effects regarding risk for rPSC, if any.

In conclusion, in this international multicentre study in PSC-prevalent countries, we show that the incidence of rPSC is higher in individuals with both pre- and post-LT inflammatory active processes. Colectomy before LT does not seem to reduce the risk of rPSC development significantly. Systemic treatment of the underlying inflammatory state caused by the autoimmune disease may be promising in the prevention of rPSC after LT.

## Financial support

The authors have declared no funding.

## Authors’ contributions

Study concept and design: TV, NE, HM, JIJ, SDM. Acquisition of data: JH, JE, NS, AG, FH, RP, BH, IA. Statistical analysis of data: NE. Interpretation of data: TV, NE, HM, JIJ, SDM. Drafting of the manuscript: TV, NE, HM, JIJ, SMD. Critical revision of the manuscript for important intellectual content: JH, JE, NS, AG, FH, RP, BH, IA. Approved the final version: all authors.

## Data availability statement

The data that support the findings of this study are available from the authors upon reasonable request and with permission of the 6 participating transplant centres.

## Conflicts of interest

The authors who have taken part in this study declared that they do not have any conflict of interest with respect to this manuscript.

Please refer to the accompanying ICMJE disclosure forms for further details.
